# Renal Cell Carcinoma: A Study through NMR-Based Metabolomics Combined with Transcriptomics

**DOI:** 10.3390/diseases4010007

**Published:** 2016-01-22

**Authors:** Rosa Ragone, Fabio Sallustio, Sara Piccinonna, Monica Rutigliano, Galleggiante Vanessa, Silvano Palazzo, Giuseppe Lucarelli, Pasquale Ditonno, Michele Battaglia, Francesco Paolo Fanizzi, Francesco Paolo Schena

**Affiliations:** 1 Consorzio C.A.R.S.O., Centro di Addestramento e Ricerca Scientifica in Oncologia, Strada Provinciale Casamassima Km 3, Valenzano (Bari) 70010, Italy; rosaragone@yahoo.it (R.R.); fabio.sallustio@uniba.it (F.S.); sara.piccinonna@libero.it (S.P.); fp.fanizzi@unisalento.it (F.P.F.); 2 Department of Emergency and Organ Transplantation, University of Bari, Bari 70124, Italy; monica.rutigliano@virgilio.it (M.R.); vanessa.galleggiante@libero.it (G.V.); palazzo@urologia.uniba.it (S.P.); giuseppe.lucarelli@inwind.it (G.L.); ditonno@urologia.uniba.it (P.D.); michele.battaglia@uniba.it (M.B.); 3 Dipartimento di Scienze e Tecnologie Biologiche ed Ambientali, Università del Salento, Prov.le Lecce-Monteroni, Lecce 73100, Italy

**Keywords:** RCC, NMR-based metabolomics, urine, transcriptomics, tumoral renal stem cells

## Abstract

Renal cell carcinoma (RCC) is a heterogeneous cancer often showing late symptoms. Until now, some candidate protein markers have been proposed for its diagnosis. Metabolomics approaches have been applied, predominantly using Mass Spectrometry (MS), while Nuclear Magnetic Resonance (NMR)-based studies remain limited. There is no study about RCC integrating NMR-based metabolomics with transcriptomics. In this work, ^1^H-NMR spectroscopy combined with multivariate statistics was applied on urine samples, collected from 40 patients with clear cell RCC (ccRCC) before nephrectomy and 29 healthy controls; nine out of 40 patients also provided samples one-month after nephrectomy. We observed increases of creatine, alanine, lactate and pyruvate, and decreases of hippurate, citrate, and betaine in all ccRCC patients. A network analysis connected most of these metabolites with glomerular injury, renal inflammation and renal necrosis/cell death. Interestingly, intersecting metabolites with transcriptomic data from CD133+/CD24+ tumoral renal stem cells isolated from ccRCC patients, we found that both genes and metabolites differentially regulated in ccRCC patients belonged to HIF-α signaling, methionine and choline degradation, and acetyl-CoA biosynthesis. Moreover, when comparing urinary metabolome of ccRCC patients after nephrectomy, some processes, such as the glomerular injury, renal hypertrophy, renal necrosis/cell death and renal proliferation, were no more represented.

## 1. Introduction

Kidney cancer is among the 10 most frequently occurring cancers in Western communities (approximately 273,000 new cases each year diagnosed worldwide, of which 102,000 in Europe). Approximately 90% of all kidney cancers are renal cell carcinomas (RCC). Smoking, overweight, obesity and germline mutations in specific genes are established risk factors for RCC. Hypertension and advanced kidney disease, which makes dialysis necessary, also increase RCC risk [1].

This tumor is morphologically and genetically heterogeneous and symptoms often occur relatively late in the disease progression. The prognosis is also variable; metastatic or recurrent RCC usually indicate poor prognosis with rare long-term survival [2,3,4]. Thus, there is a huge interest in identifying RCC specific biomarkers, early and easily detectable or able to assess the response to the therapy strategies [5].

The “-omics” technologies, genomics, proteomics, and metabolomics, have a great potential to extensively and deeply describe the response of living systems to intrinsic or extrinsic perturbations at levels of genes, proteins and metabolites [6]. Some studies have investigated RCC using omics technologies as gene expression, copy number and/or methylation analyses [7,8,9,10,11]. An extensive integrative analysis using these technologies has also been performed recently on RCC [12]. These omics studies led to the identification of pathways and biological processes involved in the RCC, such as PI3K-AKT-mTOR signaling, the p53-related pathways and mRNA processing. Moreover, various studies have been undertaken to search for candidate protein markers in RCC [13,14].

Metabolomics based on Nuclear Magnetic Resonance spectroscopy (NMR) of biofluids allows the simultaneous measurement of endogenous metabolites, reflecting the biochemical fingerprint of the organism. Multivariate statistical analyses applied on spectroscopic data aid to point out metabolic signatures of a specific status (pathology or treatment), helping to find appropriate biomarkers. Urine represents the ideal biofluid for investigating kidney dysfunctions; it can be collected frequently, easily, and noninvasively, and requires minimal sample preparation [15,16,17,18].

RCC diagnosis has been already approached using metabolic profiling of tissue, serum, and urine samples, predominantly through Mass Spectrometry (MS) measurements. 

RCC metabolic signature is characterized by alterations in metabolites related to energy metabolic pathways, particularly glycolysis, amino acid and fatty acid catabolism that are important for cell proliferation [16,19,20,21]. However, NMR-based studies remain limited. To our knowledge, there are no studies that integrate NMR-based metabolomic data with transcriptomic data obtained from clear cell RCC (ccRCC) patients. In the present study, ^1^H-NMR spectroscopy was used to examine the metabolome profile of urine samples in order to search alterations specific of patients with ccRCC compared to healthy controls; samples from some patients one-month after nephrectomy were also collected. Spectra were analyzed using multivariate statistical analysis techniques such as Principal Component Analysis (PCA) and Orthogonal Partial Least Squares (OPLS-DA). Then, metabolomic data were integrated with transcriptomic data obtained from CD133+/CD24+ cancer stem cell population of ccRCC that shared some stem cell-like features, including *in vitro* self-maintenance and differentiating capabilities. They can also regenerate tumor cells *in vitro* and induce angiogenesis *in vivo* [22].

## 2. Experimental Section

### 2.1. Sample Collection

Midstream sample of the second morning urine samples were collected from 40 ccRCC patients before partial nephrectomy (ccRCC-BN), 9 RCC patients after nephrectomy (ccRCC-AN) and 29 healthy controls (HC). Patients and controls were in the age range of 62 ± 11.6 and 56 ± 5.8 years, respectively, at the time of the study (Table 1). Of the 40 patients, 18 had a histological renal grading of G1, 11 had a grading of G2 and 11 of G3, according to the Fuhrman grade classification. Healthy subjects (HC) were males and females (21 and 8, respectively, at the time of the study) with no declared pathological condition and negative urine analysis.

Urine samples were collected in a sterile pot. The pots were stored at 4 °C until transportation to the laboratory for processing. Then, the samples were pre-treated by centrifugation at 6000× *g* for 10 min; 1.5-mL aliquots were prepared and frozen in −80 °C freezers within 5 h from urine collection.

**Table 1:** Characteristics (number, sex, age, histological classification) of patients and healthy controls, whose urine samples were provided. ccRCC-BN = patients with ccRCC before nephrectomy, RCC-AN = ccRCC patients after nephrectomy, HC = healthy controls.

	ccRCC-BN	ccRCC-AN	HC
Number	40	9	29
Male/Female	27/13	7/2	21/8
Age (years)	62.35 ± 11.65	64.11 ± 10.91	56 ± 5.81
Histological classification	18/40 G1	6/9 G1	
	11/40 G2	3/9 G2	
	11/40 G3	0/9 G3	

### 2.2. Sample Preparation

Frozen samples were thawed at room temperature and shaken before use. Aliquots of each urine sample (630 μL) were added to 70 μL of potassium phosphate buffer (1.5 M K_2_HPO_4_ in 100% ^2^H_2_O, pH 7.4), to minimize variations in metabolite NMR chemical shifts arising from differences in urinary pH, also containing 0.1% sodium 3-(trimethylsilyl)-(2,2,3,3-^2^H_4_)propionate (TSP) and 2 mM sodium azide. Samples were centrifuged at 14,000× *g* for 5 min at 4 °C to remove any solid debris. Then, 600 μL of the supernatant were placed in a 5 mm outer diameter NMR tube. All chemicals were from Sigma (Sigma-Aldrich Corporation, St. Louis, MO, USA).

### 2.3. NMR Experiments

All measurements were performed on a Bruker Avance DRX NMR spectrometer (Bruker, Karlsruhe, Germany) operating at 500.13 MHz for ^1^H observation, equipped with a TBI 500 MHz W2 5 mm xyz-gradient and with manual systems of tuning, matching, locking and shimming. A time delay of 5 min was set between sample insertion and pre-acquisition calibrations to ensure complete temperature equilibration (300 K).

For each sample, a one-dimensional Carr–Purcell–Meiboom–Gill spin-echo (CPMG) experiment was recorded (number of scans = 128, 64 k data points, spectral width 10,000 Hz, acquisition time 3.28 s, total spin–spin relaxation delay 64 ms, solvent signal saturation during the relaxation delay). This pulse sequence filters the macromolecules (such as proteins) that might be present in the sample, avoiding broad signals and improving the quality of the NMR spectrum, due to a flat baseline and reduced overlapping [23].

The free induction decays (FIDs) were multiplied by a line broadening of 0.3 Hz before Fourier transformation, phasing, and baseline correction. All spectra were referenced to the TSP signal (δ = 0.00 ppm). NMR data were processed using TopSpin 1.3 (Bruker) and visually inspected using Amix 3.9.14 (Bruker).

### 2.4. Data Processing and Analysis

The region in the range 9.5–0.5 ppm of all CPMG spectra was segmented into rectangular intervals (buckets) of fixed 0.04 ppm width, integrated and normalized to the total area (to minimize differences in urine concentration between samples) using Amix 3.9.14 (Bruker). The 6.00–4.50 ppm region comprising the signals of urea and water was excluded because of the variability in the suppression of water signal and the fluctuations in the partial cross-solvent saturation of urea signal due to solvent-exchanging protons. The bucket-table was scaled applying Pareto scaling.

### 2.5. Multivariate Statistical Analyses of ^1^H-NMR Data and Metabolite Identification

Firstly, the spectral dataset was subjected to an unsupervised (*i.e.*, without any *a priori* assumption) multivariate statistical analysis, Principal Component Analysis (PCA), to get an overview of the metabolic profiles and to highlight possible outliers. Then, it was inspected using a supervised multivariate statistical analysis, Orthogonal Partial Least Squares-Discriminant Analysis (OPLS-DA). OPLS-DA divides the systematic variation in the X-block (an input dataset of variables) into two model parts: a “predictive” part that models the co-variation between the measured data of X variable (the bucket intensities) and the response of Y variable within the groups, and an “orthogonal” part that captures systematic variation in X that is unrelated to Y. When considering a binary Y variable (in our case, binary variables of disease status) and the same number of model components, OPLS-DA performs like PLS-DA, but facilitates graphic visualization of differences and/or similarities between groups. The quality of OPLS models was described by the goodness of fit (R^2^) and the predictability based on the fraction correctly predicted in one-seventh cross-validation (Q^2^). In the seven-fold cross-validation step, seven models were built with exactly one-seventh of the data excluded from each model and each sample excluded a single time. The ability of the models to predict those samples not involved in the modeling provided a measure of the overall predictive ability of the metabolite profiling. Using these values (YPredCV), we generated a confusion matrix and calculated the specificity (true negative rate), the sensitivity (true positive rate), the accuracy (correct prediction rate), and the Cohen’s Kappa (a statistical measure of inter-rater agreement for qualitative/categorical items) at a significance level of 0.05 [24]. PCA and OPLS-DA were carried out using Simca 13 software (Umetrics, Umea, Sweden).

Metabolite identification was done using a free available database (NMR suite 8.1, Chenomx Inc., Edmonton, AB, Canada).

### 2.6. Metabolic Pathway Analysis

For the metabolic pathway analyses, we focused on unambiguously attributed metabolites having values of pq (OPLS-DA loading vector of the predictive component) normalized to unit length >|0.01| at default significance level of 0.05.

We obtained information about relative metabolite concentrations comparing the intensity of the corresponding signals in the different samples: one selected peak for each metabolite was integrated and normalized to the total area (using Amix 3.9.14), then divided for the number of protons giving rise to it. We did not use the 0.04 ppm-sized bucket intensities since, in the presence of some misalignments, the simple rectangular bucketing often makes edges of buckets cut individual peaks and single buckets contain parts of signals from different metabolites. Significance of quantitative differences for each metabolite was calculated by *t*-test.

To assess biological relationships among genes and metabolites, we used the Ingenuity Pathway Analysis software (IPA, Ingenuity System, Redwood City, CA, USA; http://www.ingenuity.com). IPA computes a score for each network according to the fit of the set of supplied focus metabolites (here, metabolites differently expressed in ccRCC patients). These scores indicate the likelihood of focus metabolites to belong to a network *versus* those obtained by chance. The canonical pathways generated by IPA are the most significant for the uploaded data set. The Benjamini–Hochberg multiple testing correction method was used to calculate the significance of the networks and functional categories.

## 3. Results and Discussion

### 3.1. Results

#### 3.1.1. Multivariate Statistical Analysis of Urine NMR Profiles

^1^H-NMR spectra were recorded for urine samples collected from 40 patients and 29 controls. In CMPG experiments, the broad signals of macromolecules (such as proteins, which might be in the sample) were removed. All spectra were aligned using the TSP peak at 0.00 ppm as reference, obtaining a good peak correspondence among samples.

**Figure 1:**
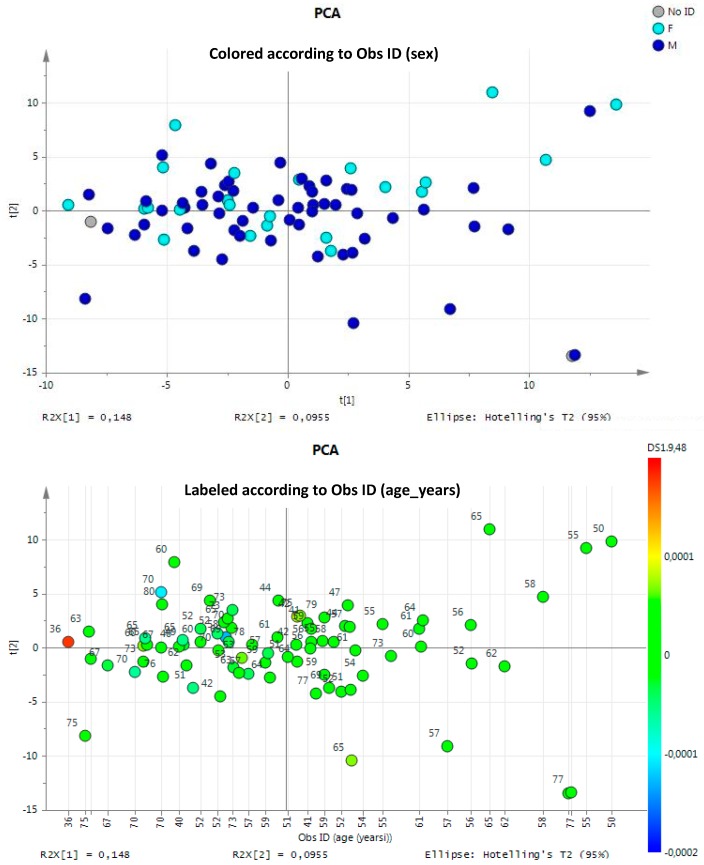
Principal Component Analysis (PCA) applied on all urine samples. It showed no clusters relating to sex (F = female, M = male) or age (years).

The digitalized CPMG spectra, segmented into rectangular 0.04 ppm-sized buckets as described above, were analyzed by PCA (unsupervised approach) to investigate general sample distribution and to identify potential outliers. PCA, usually used to get an overview of data without any *a priori* assumption, did not reveal any clustering/trend according to gender or age. We excluded any confounding effect in our study due to these variables (Figure 1) [25]. Furthermore, PCA models displayed no separation of the data-reduced profiles according to health status along the first PCs as a consequence of the high inter-individual variability typical of urine metabolome (data not shown).

Hence, the dataset was examined by OPLS-DA (a supervised approach), comparing the conditions pairs of the three groups available, *i.e.*, healthy controls (HC), ccRCC-BN patients, and ccRCC-AN patients. We obtained a good discrimination between ccRCC patients and HC (cumulative R^2^ 0.76, cumulative Q^2^ > 0.61), that revealed the existence of differences in urinary metabolome profiles (Table 2).

The intra-class variance was confirmed to be huge (the orthogonal variance was greater than the predictive one). Models built with data from the samples collected before and after nephrectomy from the same patients were quite descriptive but not predictive, having R^2^(cum) ~0.69 and Q^2^(cum) < 0, likely because of the low number of samples (Table 2).

**Figure 2:**
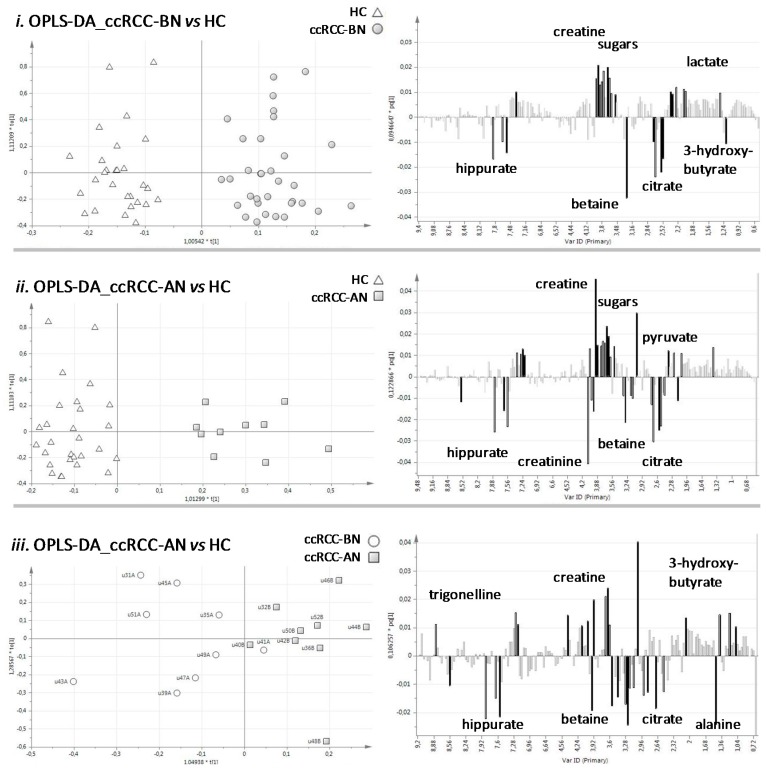

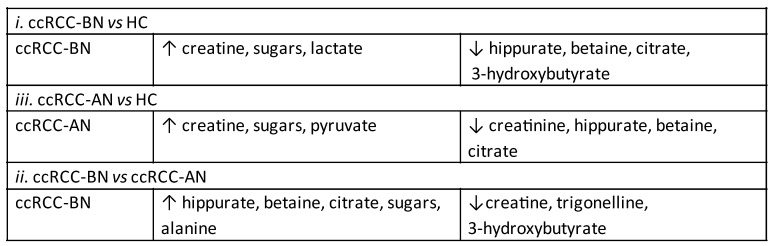
Results of OPLS-DA applied on the three groups of urine samples, compared two-by-two. On the left there are the score plots, reporting the predictive component on X-axis (t1) and the first orthogonal component on Y-axis (to1); on the right there are the loading plots, reporting the variables (buckets of 0.04 ppm) on X-axis and the corresponding loading vector values of the predictive component (pq1 normalized to unit length) on Y-axis.

In details, OPLS-DA comparisons revealed that: (i) comparing urine of ccRCC-BN patients *vs*. HC, we found in patients higher levels of creatine and lactate and lower levels of citrate, hippurate, betaine, and 3-hydroxybutyrate; (ii) comparing urine samples of ccRCC-AN patients *vs.* HC, higher levels of creatine and pyruvate and lower levels of creatinine, hippurate, betaine, and citrate were found in ccRCC-AN patients; and (iii) comparing urine samples of ccRCC-BN *vs.* ccRCC-AN patients, we found higher levels of citrate, hippurate, betaine, and alanine and lower levels of creatine, trigonelline, and 3-hydroxybutyrate before nephrectomy. Moreover, we found that the content of sugars (mainly glucose and fructose) was greater in all ccRCC patients compared to controls (Figure 2).

**Table 2:** Parameters describing the three Orthogonal Partial Least Squares-Discriminant Analysis (OPLS-DA) models obtained using SIMCA software: R^2^X(cum) (cumulative R^2^X, X variance explained by the current components), R^2^(cum) (cumulative R^2^, goodness of fit), and Q^2^(cum) (cumulative Q^2^, Q^2^ up to the specified component, where Q^2^ is the fraction of Y variation predicted by the X model in a component, according to cross-validation), specificity, sensitivity, accuracy, and Cohen’s K calculated in cross-validation.

OPLS-DA	ccRCC-BN *vs.* HC			ccRCC-AN *vs.* HC			ccRCC-BN *vs.* ccRCC-AN		
Component	R^2^X(cum)	R^2^(cum)	Q^2^(cum)	R^2^X(cum)	R^2^(cum)	Q^2^(cum)	R^2^X(cum)	R^2^(cum)	Q^2^(cum)
Model	0.474	0.765	0.615	0.429	0.874	0.685	0.256	0.699	−0.267
Predictive	0.077	0.765	0.615	0.106	0.874	0.685	0.102	0.699	−0.267
P1	0.077	0.765	0.615	0.106	0.874	0.685	0.102	0.699	−0.267
Orthogonal in X	0.397	0		0.323	0		0.155	0	
O1	0.305	0		0.234	0		0.155	0	
O2	0.397	0		0.323	0				
Specificity	0.893			0.964			0.778		
Sensitivity	0.939			0.9			0.444		
Accuracy	0.918			0.947			0.611		
Cohen’s K in cross-validation	0.835			0.864			0.222		

When observing the spread of the samples RCC-BN *vs.* HC along the two orthogonal components of the OPLS-DA model that were unrelated to the *a priori* set class (*i.e.*, disease status), no separation of them according to gender was evident. The same was observed along the minor components (the second one and the third one) of the corresponding PLS-DA model (we considered only models with ≤3 components to not over-fit the data; Table 2) (Supplementary Figure S1).

We repeated the statistical analyses considering only the males, and we obtained similar class separation and loading patterns. Moreover, when we used the females as a testing group to validate the OPLS-DA model RCC-BN *vs.* HC built with all males, their disease status was predicted with few errors (specificity 0.92, sensitivity 0.87, Cohen’s Kappa in prediction 0.8, at significance level of 0.05) (Supplementary Table S1).

All these observations confirmed that gender had no particular influence on group discrimination.

#### 3.1.2. Network Analysis

In order to investigate the role of metabolites found specifically increased in urine of ccRCC patients in the context of the pathology, we performed a bioinformatic analysis of the pathways and networks of metabolites. We integrated metabolites with data from a variety of experimental platforms and algorithmically assembled networks with other metabolites and genes on the basis of their functional and biological connectivity.

For these kinds of analyses, we focused on fifteen urinary metabolites, safely identified in all spectra and resulted to be present at significantly different levels in the compared groups (Table 3 and Supplementary Figure S2).

**Table 3:** Urinary metabolites considered for the pathway analysis and positions in the ^1^H-NMR spectrum of the corresponding selected peaks.

Metabolites	ppm
Trigonelline	9.13
Hippurate	7.84
*N*-phenylacetylglycine	7.40
Sucrose	5.25
Glucose	4.65
Creatinine	4.06
Creatine	3.93
Glycine	3.58
Carnitine	3.23
Betaine	3.28
Citrate	2.55
Pyruvate	2.35
Alanine	1.49
Lactate	1.34
3-hydroxybutyrate	1.20
3-hydroxyisobutyrate	1.37

**Figure 3:**
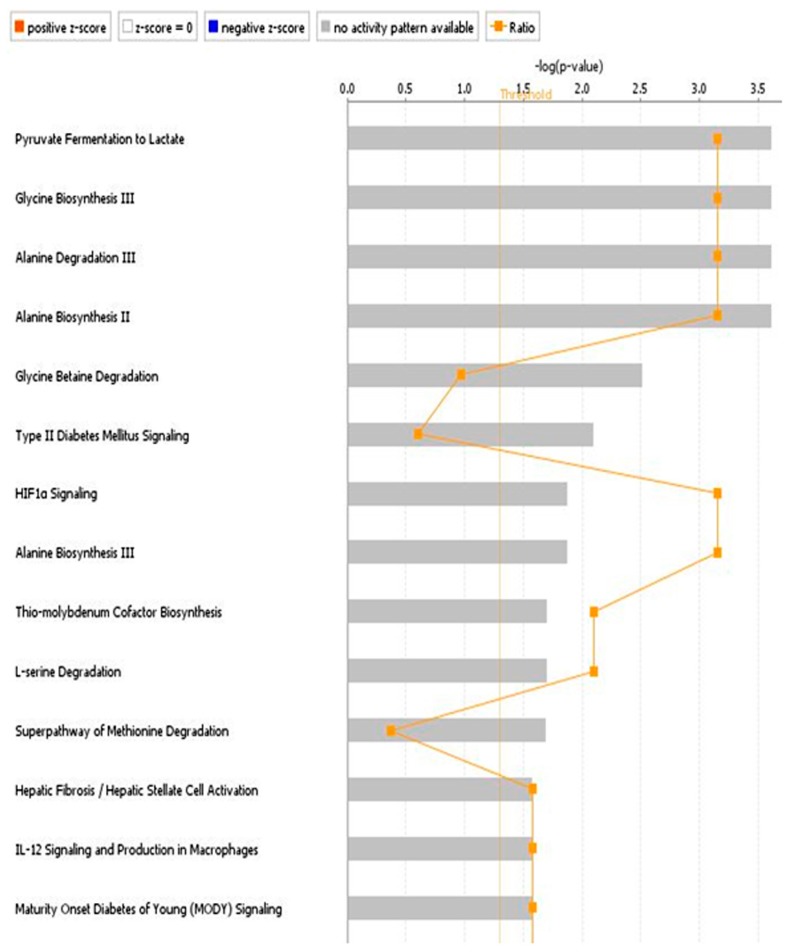
Pathways most differentially modulated in ccRCC patients generated from urinary metabolites obtained through variable size bucketing.

For each identified network, a score was computed according to the fit of the set of metabolites differently modulated in urine of ccRCC patients. These scores indicated the likelihood of identified metabolites belonging to a network *versus* those obtained by chance. Among biological pathways most differentially modulated in ccRCC patients compared to HC, we found Pyruvate Fermentation to Lactate, Glycine Biosynthesis, Alanine Biosynthesis and Degradation, Glycine Betaine Degradation and HIF1α Signaling (Figure 3 and Supplementary Table S2).

**Figure 4:**
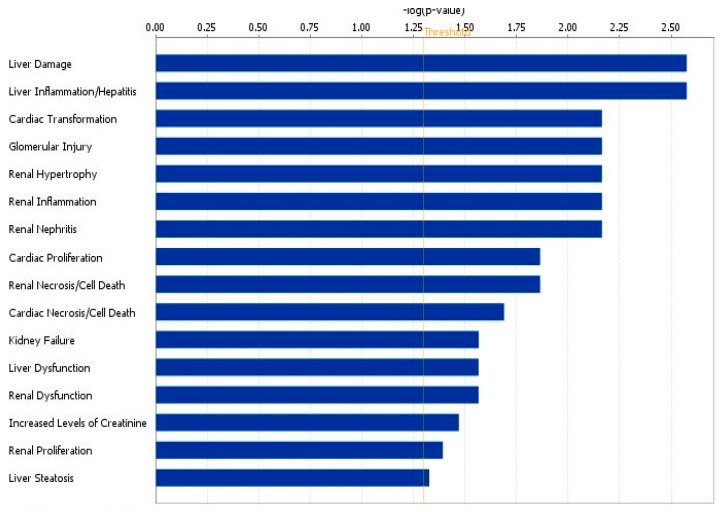
Biological processes most differentially modulated in ccRCC patients generated from urinary metabolites obtained through variable size bucketing.

Interestingly, many metabolites were associated with functions involving renal diseases and dysfunctions, such as glomerular injury, renal inflammation and renal necrosis/cell death (Figure 4 and Supplementary Table S3).

**Figure 5:**
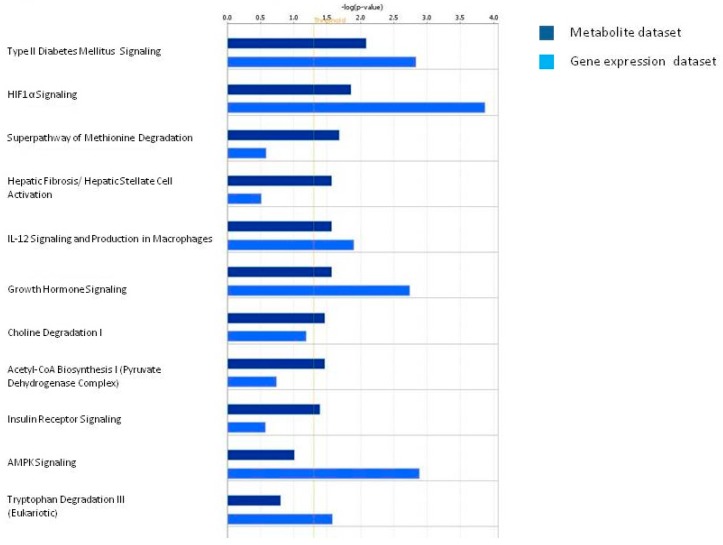
Pathway comparative analysis performed intersecting urinary metabolite dataset with the gene expression dataset from CD133+/CD24+ cancer renal stem cells of ccRCC patients.

Then, we performed a comparative analysis intersecting metabolite data with the gene expression data from CD133+/CD24+ cancer renal stem cell of ccRCC previous published [20]. Interestingly, we found functional associations of pyruvate with the HIF-α signaling and the acetyl-CoA biosynthesis. In addition, the methionine degradation, the choline degradation, and the AMPK signaling pathways resulted differentially modulated in patients both at gene expression level and at urinary metabolite level in cancer stem cells (Figure 5).

**Figure 6:**
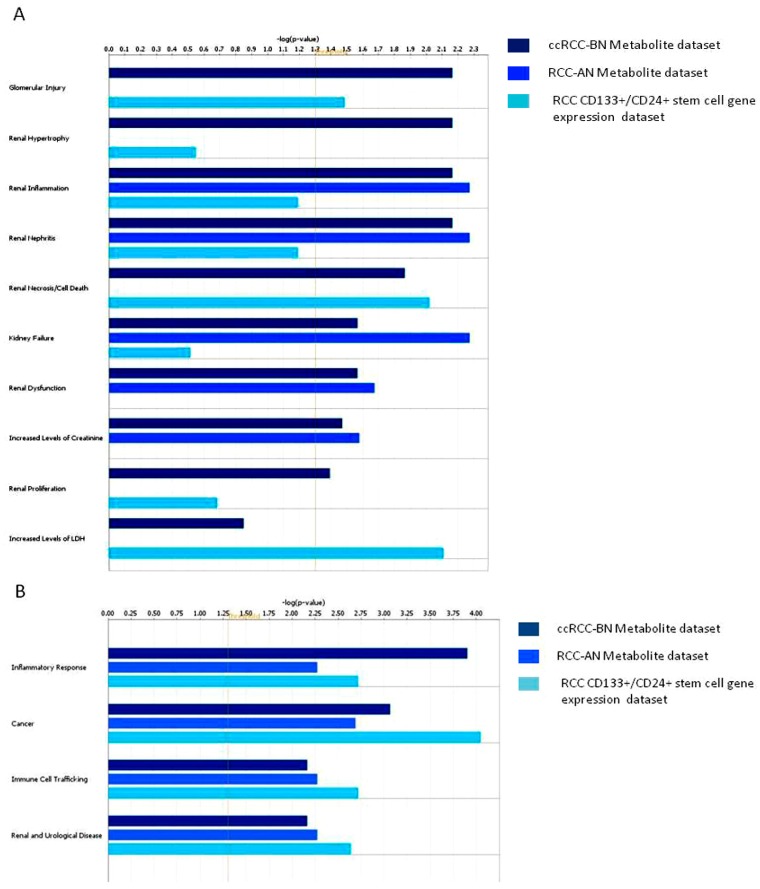
Comparative analysis of biological functions (**A**) and biological processes (**B**) generated from urinary metabolites modulated in ccRCC patients before and after nephrectomy and from gene differentially expressed in CD133+/CD24+ stem cells of ccRCC. Dark-blue bars represent ccRCC-BN urinary metabolite dataset, blue bars represent urinary metabolite dataset of ccRCC-AN and light-blue represent RCC CD133+/CD24+ stem cell gene expression dataset.

Moreover, pyruvate, betaine, and creatinine seemed to be involved in several renal dysfunctions, such as renal hypertrophy, renal inflammation, glomerular injury (Figure 6A and Supplementary Table S4), and in several biological functions, such as renal and urological disease, cancer, inflammatory response, and immune cell trafficking (Figure 6B and Supplementary Table S5). When we also compared urinary metabolome profile data of patients with ccRCC after nephrectomy, we found that after nephrectomy some biological functions were no more represented. In particular, the processes of glomerular injury, renal hypertrophy, renal necrosis/cell death and renal proliferation were no modulated by metabolites collected after nephrectomy (Figure 6).

### 3.2. Discussion

In the current study, ^1^H-NMR-based approach was applied to investigate the urinary metabolome profile in patients with ccRCC before and after nephrectomy and, for the first time, NMR data were integrated with transcriptomic data from CD133+/CD24+ cancer stem cells of patients with ccRCC.

To prevent disturbing effects due to the presence of proteins in our urine samples, we used a specific NMR pulse sequence called CPMG. Reduced spectral data were analyzed by multivariate statistical analysis. PCA suggested no influence of gender and age on our study, but indicated a significant inter-individual variability, as expected. OPLS-DA permitted a good class separation, suggesting the potential presence of distinctive metabolome features.

It seems that in ccRCC patients compared to healthy subjects levels of creatine, alanine, lactate and pyruvate increased and that levels of hippurate, citrate, and betaine decreased. When urine samples of patients before and after nephrectomy were compared, ccRCC-BN urinary samples resulted to have a greater content of hippurate, betaine, lactate and alanine. Instead, RCC-AN samples showed higher levels of creatine and pyruvate.

In order to deeply investigate the connection with the pathology of metabolites found altered in ccRCC urine samples, we performed an analysis of potentially related genes. Spectral data were used to carry out the pathway analyses.

Creatine and its by-product creatinine are metabolized in muscles (95%), kidneys, liver and pancreas. It plays a fundamental energetic role: phosphocreatine gives a phosphoric group to ADP to form ATP. Augmented urinary levels of creatine normally occur in hyper-catabolic situations, such as accelerated catabolism of tumor cells and convalescence after a surgical operation [26,27]. It could explain the high creatine excretion in urine samples collected from ccRCC patients before and after the nephrectomy. However, in the former creatine might be released by renal cells, while in the latter predominantly by muscle tissue. Serum creatinine is frequently used as a kidney dysfunction biomarker.

Hippurate is normally found in human urine at concentration depending on the microbial activity in the colon and on the individual diet [28,29]; we can speculate that its decrease may indicate a metabolic alteration and an impaired secretion at the level of the proximal tubule [30].

Citrate gives a measure of the tubular cell function [31]. We can hypothesize that reduced excretion of citrate in ccRCC patients could be likely due to the presence of a mild metabolic acidosis, and/or to an incorrect activity of the sodium/citrate co-transporter in the kidney (influenced by pH) [32]. Lower levels of hippurate and citrate were also observed in IgA nephropathy and other glomerulonephritides [33,34].

A number of studies have demonstrated that malignant transformation is associated with an increase in glycolytic flux and in anaerobic and aerobic cellular lactate excretion. Lactate seems to activate the hyaluronan synthesis by tumor-associated fibroblasts, to upregulate VEGF and HIF-1α, and to directly enhance the cellular motility [35]. It was also found in abnormal urinary concentration in patients with autosomal dominant polycystic kidney disease and glomerulonephritis [34,36]. Our data showed that lactate and glucose (from which lactate derived) were found more abundant in ccRCC-BN urine samples than in ccRCC-AN ones.

Betaine (or *N*,*N*,*N*-trimethylglycine) is a methylamine that functions as a methyl donor in essential biochemical processes, for example detoxification reactions; it plays a role in osmoregulation of most tissues. It derives through NAD-dependent dehydrogenization of betaine aldehyde, primarily formed through the mitochondrial oxidation of choline in the liver and kidney [37]. Betaine can protect the kidneys from damage; it occurs abundantly in the medulla, where (as other osmolytes) it acts as a chemical chaperone, countering the denaturing effect of urea on proteins. Betaine re-methylates homocysteine to methionine by betaine homocysteine methyltransferase (BHMT), an enzyme osmoregulated [38]. It is likely that the production of reactive and toxic compounds, plausibly due to the inflammation response activated in hypoxic conditions within the tumor mass, could stimulate detoxification reactions and consume betaine reserves. This could explain why ccRCC-BN samples showed lower content of betaine. In ccRCC-AN, low betaine levels may be linked to detoxification from drugs or to insufficient production of betaine.

Then, we performed a comparative analysis intersecting metabolite data with the gene expression data obtained from CD133+/CD24+ renal cancer stem cells of ccRCC previous published [22].

We found both genes and metabolites belonging to HIF-α signaling, methionine and choline degradation and the acetyl-CoA biosynthesis.

The modulation of the HIF-α signaling pathway is interesting since this pathway is implicated with the angiogenesis that is one of the most important hallmarks of RCC. Tumoral CD133+/CD24+ stem cells were also able to trigger an angiogenic response. In fact, they can develop numerous allantoic vessels in the chick embryo chorioallantoic membrane. Several genes involved in cellular differentiation are directly or indirectly regulated by hypoxia and by HIF-1. These include Epo, lactate dehydrogenase A (LDHA), ET1, transferrin, transferrin receptor, VEGF, PDGF-β, FGF, and genes affecting glycolysis. HIF1-α-activated genes include VEGF, which promotes angiogenesis; GLUT1, which activates glucose transport; LDHA, which is involved in the glycolytic pathway; and Epo, which induces erythropoiesis. HIF1-α also activates transcription of NOS, which promotes vasodilation and angiogenesis [39,40,41].

Interestingly, the pyruvate, betaine and creatinine metabolites seem to be involved in several renal dysfunction, as glomerular injury, renal hypertrophy, renal inflammation and we found that, after nephrectomy, some renal dysfunction processes, as glomerular injury, renal hypertrophy, renal necrosis/cell death and renal proliferation, were no more represented. This could indicate the biochemical change after the kidney removal.

The principal limitation of this study is that even if the modulation of these metabolites is interesting and is supported by the common pathways modulated in the gene expression data, the validation of metabolites by biochemical assays is lacking. Further studies will be needed to confirm these data.

## 4. Conclusions

In summary, metabolomics based on ^1^H-NMR spectroscopy, revealed metabolic features typical of urine samples of ccRCC patients, which distinguished them from healthy individuals and patients after nephrectomy. This is due overall to differences in concentration of the same metabolites. Results from the interconnection between transcriptional data from a particular type of tumoral renal stem cells and urinary metabolomic data may add knowledge on mechanisms underlying ccRCC pathogenesis and disease progression.

## Supplementary Materials

Supplementary materials are accessible at: www.mdpi.com/2079-9721/4/1/7/s1.
