# Supplementary material for: Renal Cell Carcinoma: A Study through NMR-Based Metabolomics Combined with Transcriptomics
Source: Diseases. 2016 Jan 22;4(1):7. doi: 10.3390/diseases4010007 (PMC5456302; doi:10.3390/diseases4010007)
Supplement: Supplementary file 1 [file diseases-04-00007-s001.zip › diseases-04-00007-supplementary file 1.pdf]

Supplementary Materials: Renal Cell Carcinoma: A Study through NMR-Based Metabolomics Combined with Transcriptomics

Rosa Ragone, Fabio Sallustio, Sara Piccinonna, Monica Rutigliano, Galleggiante Vanessa, Silvano Palazzo, Giuseppe Lucarelli, Pasquale Ditonno, Michele Battaglia, Francesco Paolo Fanizzi and Francesco Paolo Schena

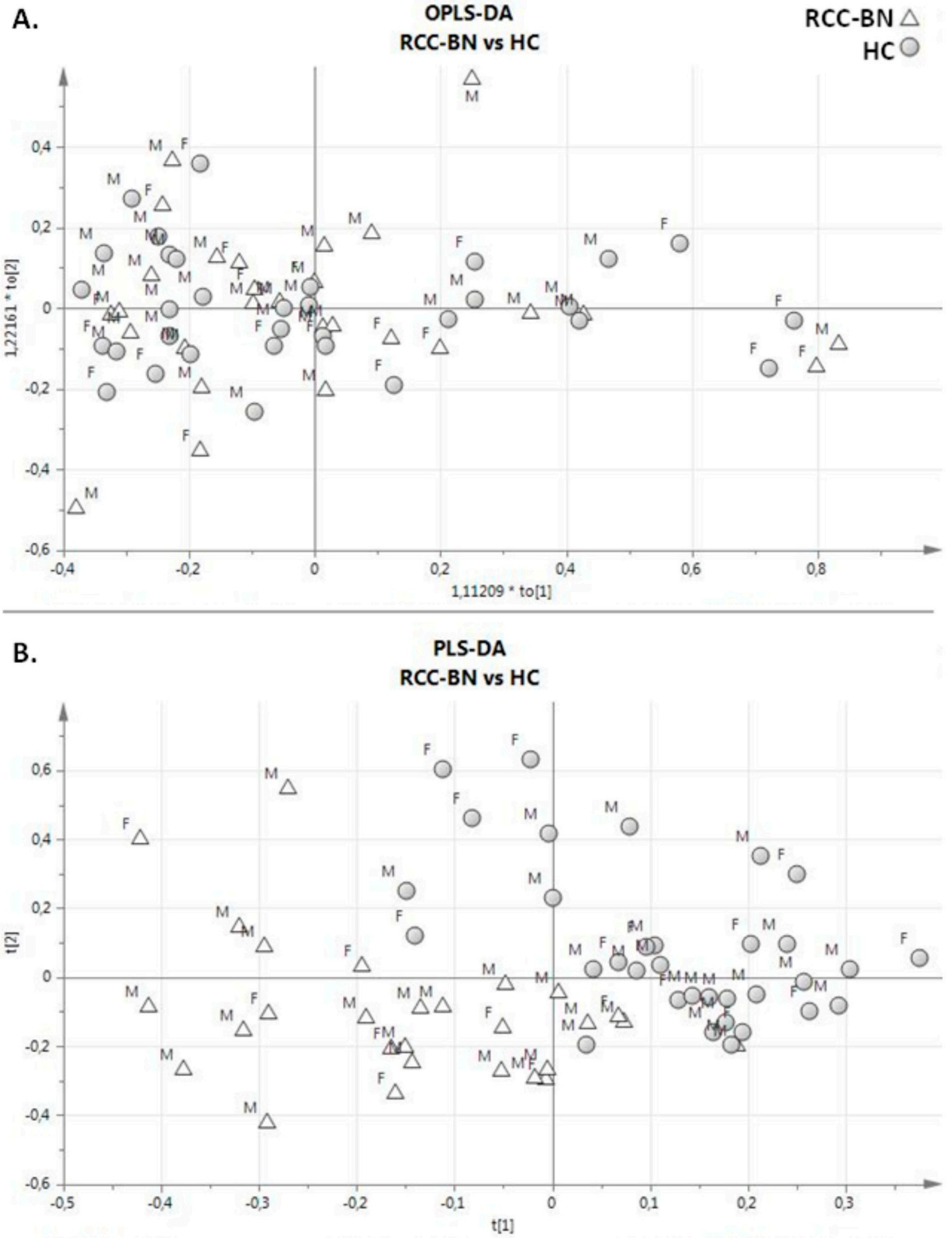

Figure S1. *Cont.*

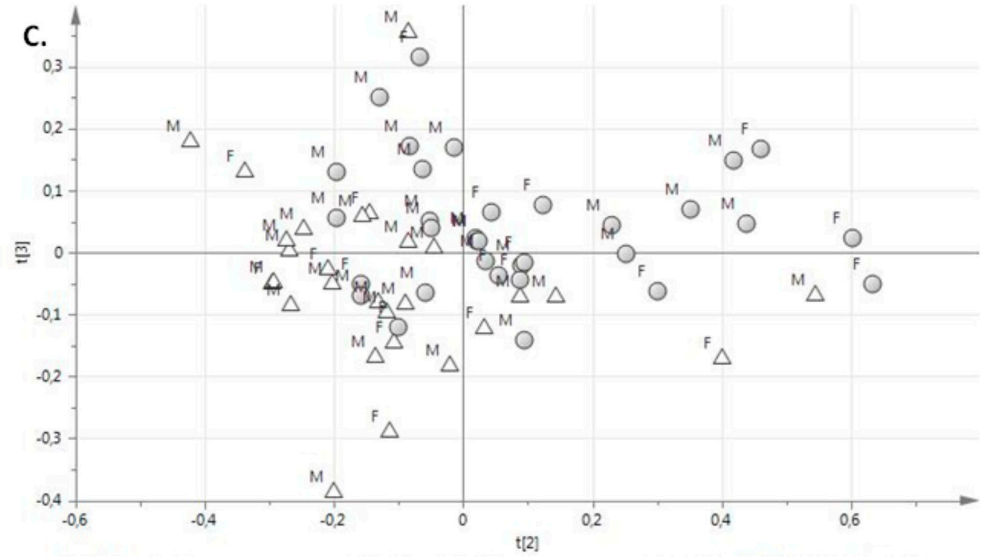

Figure S1. Score plot of OPLS-DA model for RCC-BN *vs.* HC, showing the first (to1) and the second (to2) orthogonal components (**A**). Score plots of PLS-DA model for RCC-BN *vs.* HC, showing the first (t1) *vs.* the second (t2) components (**B**) and the second (t2) *vs.* the third (t3) components (**C**). Samples are labeled with F for females and M for males. There is no grouping according to gender neither along orthogonal components in OPLS-DA (to1 *vs.* to2) nor along minor components in PLS-DA (t2 *vs.* t3).

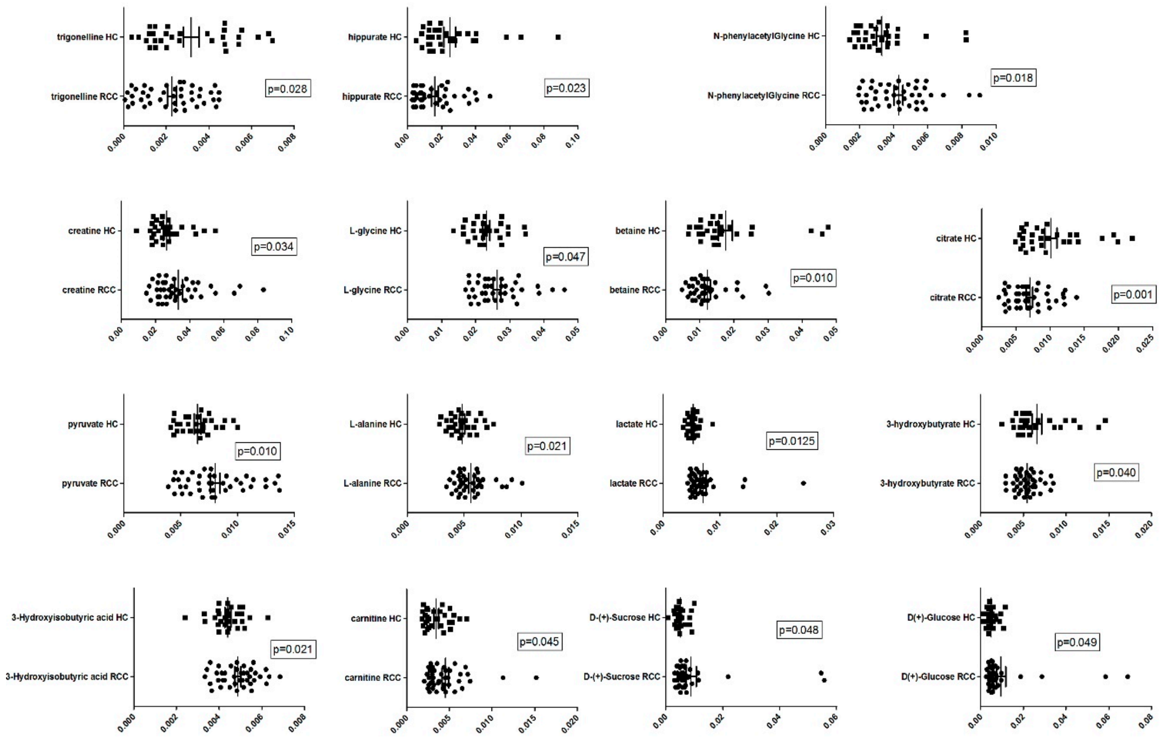

Figure S2. Quantitative differences and the *p*-value *t*-test for each metabolite calculated on the basis of the intensity of the corresponding signals in the different samples.

Table S1. Parameters describing the models (OPLS-DA and PLS-DA) built using only the spectral data of male subjects.

**ccRCC-BN *vs.* HC Only Males**							
**OPLS-DA Model**				**PLS-DA Model**			
**Component**	R^2X(cum)	R^2(cum)	Q^2(cum)	**Component**	R^2X(cum)	R^2Y(cum)	Q^2(cum)
**Model**	0.367	0.662	0.426	**1**	0.226	0.421	0.27
**Predictive**	0.112	0.662	0.426	**2**	0.367	0.662	0.452
**P1**	0.112	0.662	0.426				
**Orthogonal in X**	0.254	0					
**O1**	0.254	0					
**Specificity**	0.8			**Specificity**	0.8		
**Sensitivity**	0.905			**Sensitivity**	0.905		
**Accuracy**	0.855			**Accuracy**	0.855		
**Cohen's K in cross-validation**	0.706			**Cohen's K in cross-validation**	0.706		
